# Automated processing of whole blood samples into microliter aliquots of plasma

**DOI:** 10.1155/S1463924688000045

**Published:** 1988

**Authors:** C. A. Burtis, W. W. Johnson, W. A. Walker

**Affiliations:** 1 Chemical Technology Oak Ridge National Laboratory Oak Ridge Tennessee 37831 USA; 2 Instrumentation and Controls Oak Ridge National Laboratory Oak Ridge Tennessee 37831 USA; 3 Plant and Equipment Divisions Oak Ridge National Laboratory Oak Ridge Tennessee 37831 USA

## Abstract

A rotor that accepts and automatically processes a bulk aliquot of a single blood sample into multiple aliquots of plasma has been designed and built. The rotor consists of a central processing unit, which includes a disk containing eight precision-bore capillaries. By varying the internal diameters of the capillaries, aliquot volumes ranging 1 to 10 μl can be prepared. In practice, an unmeasured volume of blood is placed in a centre well, and, as the rotor begins to spin, is moved radially into a central annular ring where it is distributed into a series of processing chambers. The rotor is then spun at 3000 rpm for 10 min. When the centrifugal field is removed by slowly decreasing the rotor speed, an aliquot of plasma is withdrawn by capillary action into each of the capillary tubes. The disk containing the eight measured aliquots of plasma is subsequently removed and placed in a modifed rotor for conventional centrifugal analysis. Initial evaluation of the new rotor indicates that it is capable of producing discrete, microliter volumes of plasma with a degree of accuracy and precision approaching that of mechanical pipettes.


Journal of Automatic Chemistry, Vol. 10, No. 1 (January–March 1988), pp. 6–9

### Automated processing of whole blood samples into microliter aliquots of plasma

**C. A. Burtis[1], W. F. Johnson[2], and W. A. Walker[3]**

*Chemical Technology[1], Instrumentation and Controls[2], and Plant and Equipment Divisions[3], Oak Ridge National Laboratory*, Oak Ridge, Tennessee 37831, USA*

*A rotor that accepts and automatically processes a bulk aliquot of a single blood sample into multiple aliquots of plasma has been designed and built. The rotor consists of a central processing unit, which includes a disk containing eight precision-bore capillaries. By varying the internal diameters of the capillaries, aliquot volumes ranging 1 to 10 µl can be prepared. In practice, an unmeasured volume of blood is placed in a centre well, and, as the rotor begins to spin, is moved radially into a central annular ring where it is distributed into a series of processing chambers. The rotor is then spun at 3000 rpm for 10 min. When the centrifugal field is removed by slowly decreasing the rotor speed, an aliquot of plasma is withdrawn by capillary action into each of the capillary tubes. The disk containing the eight measured aliquots of plasma is subsequently removed and placed in a modified rotor for conventional centrifugal analysis. Initial evaluation of the new rotor indicates that it is capable of producing discrete, microliter volumes of plasma with a degree of accuracy and precision approaching that of mechanical pipettes.*

### Introduction

The development was previously reported of a simple rotor for automatically processing individual, unmeasured aliquots of whole blood into measured, microliter aliquots of plasma, Burtis *et al.* [1]. Evaluation of this device, designated the 'Capillary Processor and Aliquoter Model 1' (CPA-1), and its subsequent use with a centrifugal analyser, Burtis *et al.* [2], demonstrated its capability for producing aliquots of plasma with a degree of precision approaching that of a mechanical pipette. The device has numerous applications; however, it was limited to producing a batch of single aliquots of plasma from single aliquots of whole blood.

To increase versatility, a second version of the rotor has been designed, which is capable of accepting an unmeasured, bulk aliquot of whole blood and processing it into multiple, microliter aliquots of plasma. This paper describes the new version (CPA-2), explains how it functions, and presents data that demonstrate its analytical capabilities.

### Experimental

*Capillary Processor and Aliquoter, Model 2 (CPA-2)*

The CPA-2 consists of a sampling disk and a processing rotor (see figure 1).

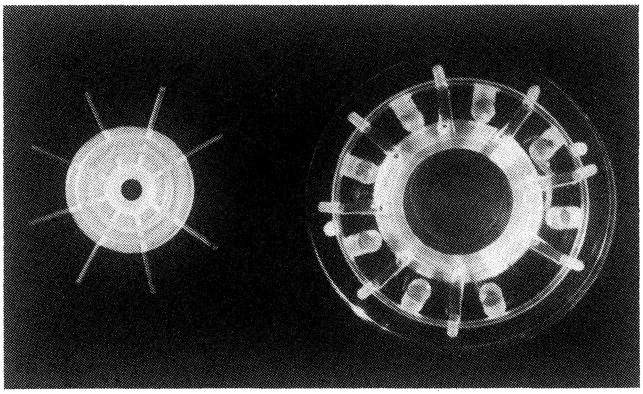

*Figure 1. Top view of the Capillary Processor and Aliquoter, Model 2 (CPA-2). An aliquot of blood is introduced into the processing rotor, and after centrifugation aliquots of plasma are drawn by capillary action into the measuring capillaries of the sampling disk.*

The sampling disk can contain up to eight capillary tubes that have been inserted in a spoke arrangement. These tubes are constructed from 2.54 cm lengths of precision-bore tubing, and specific volumes can be obtained by selecting capillaries of different internal diameters. For the purpose of these studies, capillaries having volume capacities of 1, 2, 5, and 10 µl (figure 2) were purchased from Friedrich and Dimmock, Millville, New Jersey, USA. Capillaries of the same volume, or a mixture of capillaries with different volumes, can be inserted within a given sampling disk. This capability increases the versatility of the CPA-2 so that aliquot volumes can be selected for any desired situation and can also be matched with the volumes required for specific chemical assays.

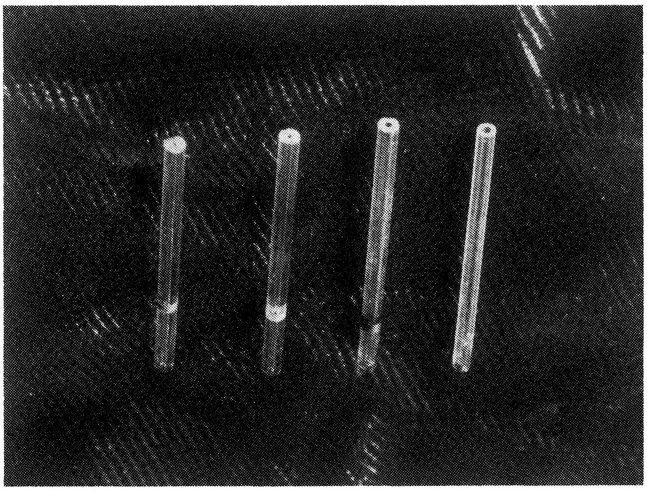

*Figure 2. Photograph of the 2·54-cm capillaries used in the CPA-2. The internal volume of the capillaries varies as a function of internal diamter. Internal volumes of the capillaries shown are 10, 5, 2, and 1 µl (left to right).*

* *Operated by Martin Marietta Energy Systems, Inc., under contract DE-AC05-840R21400 with the US Department of Energy.*

© *1988 US Government.*

The processing rotor is fabricated from an 8.5 cm disk of clear acrylic plastic that contains eight individual chambers, each of which will accept a measuring capillary (see figure 1). The processing rotor also has a central receiving chamber which connects with an annular ring. The ring interconnects with all the processing chambers and thus provides a means of apportioning and distributing a bulk aliquot of blood into individual aliquots – one per processing chamber.

The sampling disk and capillaries fit into matching chambers in the processing rotor (figure 3). The assembled CPA-2 is then placed into the rotor compartment of a centrifugal analyser (Burtis *et al.* [2]) for subsequent processing.

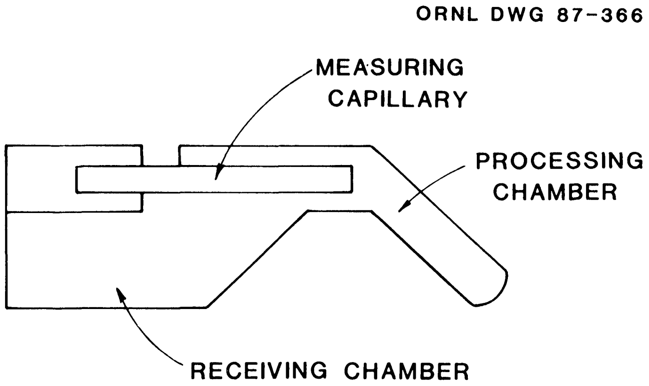

*Figure 3. Side view of one channel of the CPA-2 .*

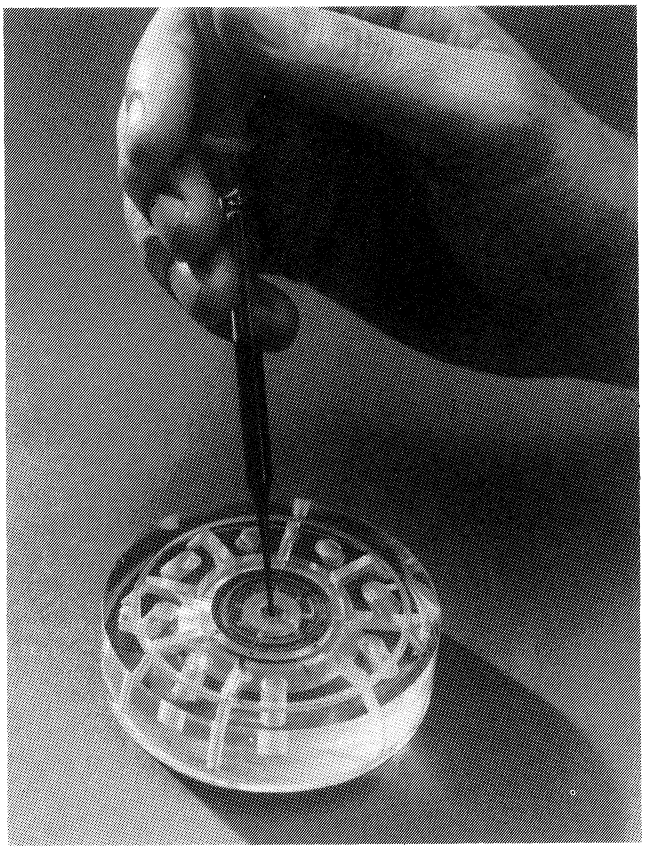

*Figure 4. Introduction of an aliquot of blood into the processing rotor of the CPA-2 .*

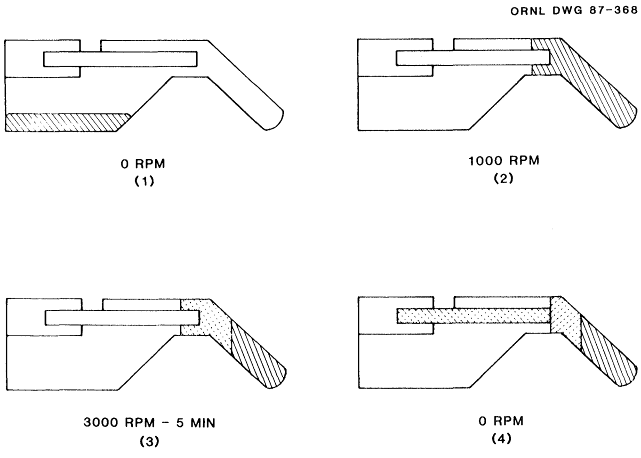

*Figure 5. Schematic representation of the process used by the CPA-2 to produce multiple aliquots of plasma from an aliquot of blood (see text for details).*

*Principle of operation*

With the rotor at rest, a 3 ml to 4 ml aliquot of whole blood is introduced into the receiving chamber of the processing rotor (figure 4), where it remains as long as the rotor is stationary. As shown schematically in figure 5, when the rotor is accelerated to 1000 rpm, the aliquot of blood is moved radially into a central annular ring where it is apportioned and distributed into the processing chambers. Increasing the rotor speed to 3000 rpm and maintaining it there for 5 min will generate a centrifugal force of 400 × g (Burtis *et al.* [3]), which separates the aliquots of plasma from the cellular components of the aliquots of blood. When the centrifugal field is removed by slowly decreasing the rotor speed to 0, an aliquot of plasma is withdrawn by capillary action into each of the measuring capillaries. The sampling disk, containing the capillaries filled with plasma, is then removed and the tip of each is carefully wiped with tissue. At this point, the disk can be placed into a modified analytical rotor for conventional centrifugal analysis, Burtis *et al.* [2], or the capillaries can be removed individually for a variety of analyses.

*Centrifugal analysis*

An analytical system based around a miniature centrifugal analyser was used in these studies. The analyser was used both as centrifuge (to process the blood samples into measured aliquots of plasma) and as a photometer (to obtain results in the evaluation studies). Details of this system and its operation have been previously published, Burtis *et al.* [1].

In a typical run, aliquots of reagent are loaded into their respective chambers in the analytical rotor by using either a mechanical pipette (Model 25004; Micromedic Systems, Inc., Horsham, Pennsylvania, USA) or a manual pipette (Model EDP; Rainin Instrument Co., Inc., Emeryville, California, USA). The sampling disk containing capillaries filled with plasma is then placed in a modified analytical rotor, Burtis *et al.* [1]. This rotor contains 17 cuvettes, one of which is a reference and the other 16 are analytical. The rotor will accept either the 16-capillary disk used in the CPA-1 or the eight-capillary disk used in the CPA-2. In practice, the CPA-2 sampling disk is inserted into the modified rotor so that the capillaries reside in the even-numbered chambers. Once the disk is in place, the analysis is initiated by transferring the contents of the reagent chambers and capillaries into their respective cuvettes. To transfer the samples and mix with reagent, the rotor is first slowly accelerated to 1000 rpm, then rapidly accelerated to 4000 rpm, and, finally, abruptly stopped with instantaneous braking. After reacceleration of the rotor to 1000 rpm, data acquisition is initiated.

*Evaluation procedures*

The analytical performance of the CPA-2 was assessed using gravimetric, photometric, and chemical techniques.

*Gravimetric and photometric evaluation* – the precision and accuracy with which the capillaries could entrain and transfer aliquots of liquid have been evaluated using both gravimetric and photometric techniques. The details of this study have been described previously, Burtis *et al.* [1].

*Chemical evalaution* – the analytical capabilities of the CPA-2 were determined by performing a set of experiments in which it was used to prepare aliquots of a reference solution of glucose, which were subsequently analysed using a Glucose-HK Endpoint Reagent Kit (SmithKline/Beckman, Carlsbad, California, USA). The reference solution was prepared by disolving 100 mg of glucose (Standard Reference Material 917, National Bureau of Standards, Washington, D.C.) in 100 ml of distilled water. Three replicate runs were conducted for each capillary volume. While the aliquots of the reference glucose were being prepared, the mechanical pipette was used to load 120-μl aliquots of glucose reagent into the reagent chambers 2 through 17. A 120-μl volume of water was dispensed into reagent chamber 1. The sampling disk was then loaded into the analytical rotor such that the capillaries resided in the even-numbered chambers. After the reactions had been initiated, they were allowed to go to completion and the resulting absorbances were measured. The glucose concentration of the contents of each even-numbered cuvette was calculated by multiplying the absorbance (corrected for the mean reagent absorbance obtained from the odd-numbered cuvettes) by the appropriate substrate factor, Tiffany *et al.* [4], that was computed for each capillary volume.

In a second set of experiments, the CPA-2 was used to prepare replicate aliquots of plasma from whole blood samples that had been collected from healthy subjects into tubes containing ethylenediamine-tetraacetic acid (EDTA) as an anticoagulant. The glucose content of the plasma contained in each capillary was determined by using the procedure described above. Several analytical runs were made for each of the capillary volumes.

### Results

*Precision and accuracy*

*Gravimetric and photometric evaluation* – in a previous study, (Burtis *et al.* [1]) it was demonstrated that the capillary tubes used in both the CPA-1 and CPA-2 were capable of entraining and delivering microliter volumes of liquids with a degree of precision and accuracy near that of mechanical pipettes. For example, the volumetric capacities of the 1 μl to 10 μl capillary tubes were within 0·5 to 6·0% of their nominal volumes computed from their physical dimensions, and their tube-to-tube precision ranged from ±0·8% for a 10 μl volume to ±3·7% for 1 μl. In addition, photometric evaluation studies demonstrated that capillary tubes and a mechanical pipette produced microliter volumes of liquid with a comparable degree of accuracy (figure 6).

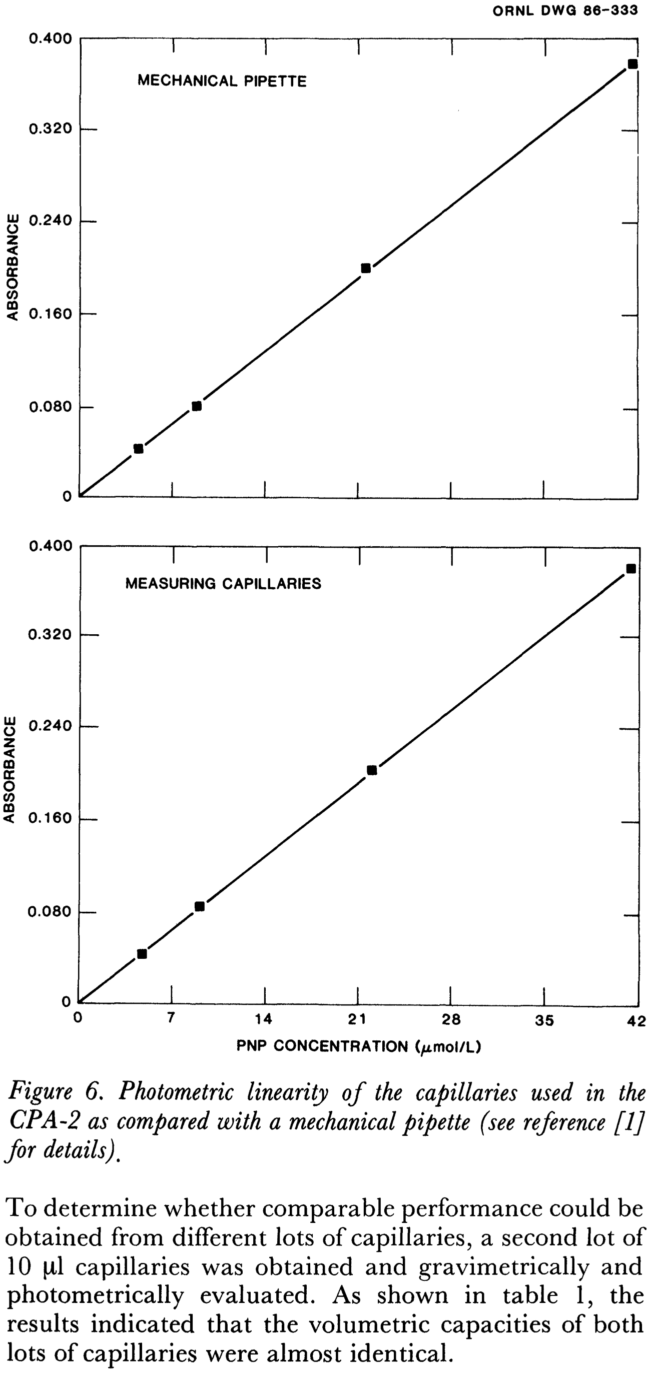

*Figure 6. Photometric linearity of the capillaries used in the CPA-2 as compared with a mechanical pipette (see reference [1] for details).*

To determine whether comparable performance could be obtained from different lots of capillaries, a second lot of 10 μl capillaries was obtained and gravimetrically and photometrically evaluated. As shown in table 1, the results indicated that the volumetric capacities of both lots of capillaries were almost identical.

*Chemical evaluation* – to demonstrate the analytical capabilities of the CPA-2, we prepared aliquots of a reference solution of glucose for each of the four capillary volumes. Analysis of these aliquots (table 2) demonstrated that the

*Table 1. Gravimetric and photometric evaluation of two different lots of 10 μl capillaries.*

| Lot number | Volume (μl)[1] Mean | Standard deviation | Relative standard deviation | Absorbance[2] Mean | Standard deviation | Relative standard deviation |
|---|---|---|---|---|---|---|
| 1 | 9·95 | 0·081 | 0·8% | 0·3803 | 0·0052 | 1·4% |
| 2 | 9·98 | 0·054 | 0·5% | 0·3754 | 0·0057 | 1·5% |

(1) $N=$ 10 observations each.
(2) $N=$ 16 observations each; 400 nm, 0·5 cm pathlength.

*Table 2. The effect of capillary volume on the processing and aliquoting capabilities of the CPA-2.*

| Capillary volume (μl) | Glucose concentration (mg/dl) N | Mean | Standard deviation | Relative standard deviation |
|---|---|---|---|---|
| 1 | 24 | 99·7 | 4·02 | 4·0% |
| 2 | 24 | 99·5 | 1·86 | 1·9% |
| 5 | 24 | 100·1 | 1·74 | 1·8% |
| 10 | 24 | 99·1 | 1·60 | 1·6% |

*Table 3. Determination of glucose in whole blood samples using the CAP-2 for processing and aliquoting.*

| Capillary volume (μl) | Sample No. | Glucose (mg/dl) Mean | Standard deviation | Relative standard deviation (%) |
|---|---|---|---|---|
| 1 | 1 | 74·5 | 1·6 | 2·2 |
| | 2 | 80·6 | 2·3 | 2·9 |
| | 3 | 70·2 | 1·6 | 2·2 |
| | 4 | 71·6 | 3·4 | 4·7 |
| 2 | 5 | 74·2 | 1·3 | 1·7 |
| | 6 | 73·1 | 1·3 | 1·8 |
| | 7 | 74·4 | 1·3 | 1·7 |
| | 8 | 72·9 | 2·7 | 3·6 |
| 5 | 9 | 81·1 | 1·1 | 1·3 |
| | 10 | 91·2 | 0·8 | 0·9 |
| | 11 | 67·7 | 1·4 | 2·0 |
| | 12 | 80·5 | 1·0 | 1·3 |
| | 13 | 87·1 | 1·8 | 2·0 |
| 10 | 14 | 84·7 | 1·6 | 1·9 |
| | 15 | 81·7 | 0·8 | 1·0 |
| | 16 | 95·6 | 1·1 | 1·1 |
| | 17 | 69·7 | 0·6 | 0·8 |
| | 18 | 71·1 | 0·4 | 0·5 |

actual capillary volumes were equivalent to the nominal volumes were within 1% of the glucose concentration of 100 mg/dl. The precision of these measurements was inversely related to volume capacity since the relative standard deviation ranged from ±4·0% for the 1 μl capillaries to ±1·6% for the 10 μl tubes.

To further demonstrate the analytical capabilities of the CPA-2, we processed a number of blood samples and analysed the resulting aliquots of plasma for glucose. Operationally, no problems were encountered, and the results indicate (table 3) that the glucose contents of blood samples could be determined with a mean precision ranging from ±3·0% for the 1 μl capillaries to ±1·0% for the 10 μl capillaries.

### Discussion

These results demonstrate that the CPA-2 can be used to process a bulk aliquot of blood into multiple aliquots of plasma with a degree of precision and accuracy comparable to that obtained with manual and mechanical pipettes. Multivolume versatility is also possible by using capillaries with different physical dimensions – and hence different internal volumes. Thus, aliquot volumes can be selected to match the volumes required for specific chemical assays.

It should be noted that the CPA-1 and the CPA-2 are complementary devices that provide the analyst with the versatility needed to meet certain analytical requirements. For example, if a large number of blood samples are analysed for a single component, the CPA-1 would be used to process the samples into single aliquots of plasma. However, if each blood sample needs to be analysed for multiple analytes, the CPA-2 would be used to prepare the multiple aliquots of plasma for subsequent analysis.

In summary, the CPA-2 can be used to prepare multiple aliquots of plasma for subsequent analysis. As demonstrated here, this device can be interfaced directly with a centrifugal analyser; together, they provide an integrated system to process and analyse single blood specimens for multiple analytes. As with the CPA-1, the CPA-2 can also be used to process liquids in several analytical situations requiring the preparation of discrete, microliter volumes of samples for subsequent analysis.

### References

1. Burtis, C. A., Johnson, W. F. and Walker, W. A., *Clinical Chemistry*, **32** (1986), 1642.
2. Burtis, C. A., Johnson, W. F., Mailen, J. C., Overton, J. B., Tiffany, T. O. and Watsky, W. B., *Clinical Chemistry*, **19** (1973), 895.
3. Burtis, C. A., Bostick, W. D. and Johnson, W. F., *Clinical Chemistry*, **21** (1975), 1225.
4. Tiffany, T. O., Jansen, J. M., Burtis, C. A., Overton, J. B. and Scott, C. D., *Clinical Chemistry*, **18** (1972), 829.

